# Efficacy of color Doppler ultrasound and contrast-enhanced ultrasound in identifying vascular invasion in pancreatic ductal adenocarcinoma

**DOI:** 10.1186/s13244-024-01779-5

**Published:** 2024-07-25

**Authors:** Wan-Ying Jia, Yang Gui, Xue-Qi Chen, Li Tan, Jing Zhang, Meng-Su Xiao, Xiao-Yan Chang, Meng-Hua Dai, Jun-Chao Guo, Yue-Juan Cheng, Xiang Wang, Jia-Hui Zhang, Xiao-Qian Zhang, Ke Lv

**Affiliations:** 1 Department of Ultrasound, Peking Union Medical College Hospital, Chinese Academy of Medical Sciences and Peking Union Medical College, Beijing, China; 2 Department of Pathology, Peking Union Medical College Hospital, Chinese Academy of Medical Sciences and Peking Union Medical College, Beijing, China; 3 Department of General Surgery, Peking Union Medical College Hospital, Chinese Academy of Medical Sciences and Peking Union Medical College, Beijing, China; 4 Department of Medical Oncology, Peking Union Medical College Hospital, Chinese Academy of Medical Sciences and Peking Union Medical College, Beijing, China; 5 Department of Radiology, Peking Union Medical College Hospital, Chinese Academy of Medical Sciences and Peking Union Medical College, Beijing, China

**Keywords:** Pancreatic ductal adenocarcinoma, Contrast-enhanced ultrasound, Color Doppler ultrasound, Vascular invasion

## Abstract

**Objectives:**

To compare color Doppler ultrasound and contrast-enhanced ultrasound (CEUS) in evaluating vascular invasion in pancreatic ductal adenocarcinoma (PDAC).

**Materials and methods:**

This retrospective study included 210 patients with PDAC who were evaluated by color Doppler ultrasound, CEUS, and contrast-enhanced computed tomography (CECT) at our institution between January 2017 and December 2020. Pathologic results were used as the gold standard in patients who underwent surgical and intraoperative exploration. For nonsurgical patients, CECT results were used as the reference standard. The vessels evaluated included those in the peripancreatic arterial system and venous system. The diagnostic performances of color Doppler ultrasound and CEUS for vascular invasion were compared.

**Results:**

In 51 patients who underwent surgery and intraoperative exploration, color Doppler ultrasound and CEUS differed only in assessing venous system invasion in patients with PDAC of the pancreatic body and tail, with the former being superior to the latter. In 159 nonsurgical patients, there was no difference between CEUS and color Doppler ultrasound in assessing superior mesenteric arteriovenous invasion. CEUS was superior to color Doppler ultrasound in evaluating the celiac artery and its branches, with an accuracy of up to 97.8% for some vessels. Color Doppler ultrasound was ideal for evaluating the splenic and portal veins.

**Conclusion:**

CEUS is more suitable for the evaluation of peripancreatic arteries than color Doppler. CEUS combined with color Doppler ultrasound can be used as a potential supplement to CECT and is also expected to be used to evaluate vascular invasion of PDAC after chemotherapy.

**Critical relevance statement:**

Contrast-enhanced US and color Doppler in the assessment of vascular invasion in pancreatic ductal adenocarcinoma have their respective advantages, through standardized ultrasound processes are expected to improve the efficiency of inspection.

**Key Points:**

- Contrast-enhanced US has unique advantages in assessing pancreatic ductal adenocarcinoma invasion of the celiac artery.
- Doppler imaging is of high value in assessing venous system invasion.
- Standardization of ultrasound imaging procedures for pancreatic ductal adenocarcinoma is expected to improve efficiency.

**Graphical Abstract:**

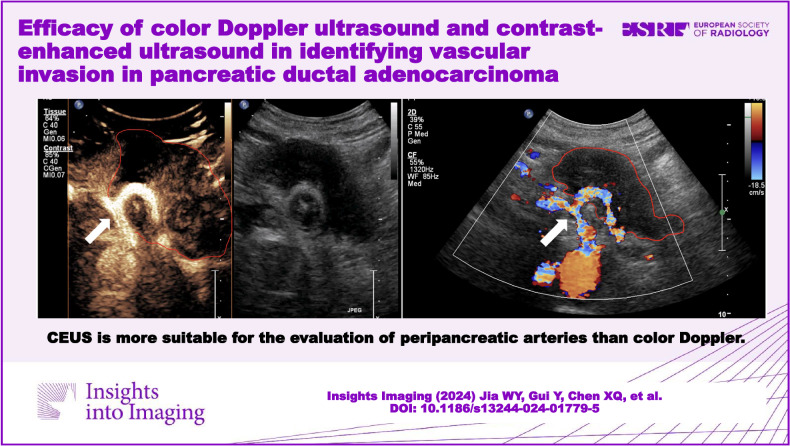

## Introduction

Recent advances in chemotherapy have improved the survival of patients with pancreatic ductal adenocarcinoma (PDAC). However, surgical resection is still the only potentially curative treatment for this terrible disease [1]. For such patients, the evaluation of vascular invasion is critical in assessing the surgical situation and evaluating chemotherapy intervals. Therefore, the accurate assessment of tumor vascular invasion has been the focus of research on pancreatic carcinoma imaging.

According to the National Comprehensive Cancer Network (NCCN) guidelines, vascular invasion is divided into the arterial and venous systems [2]. The guidelines recommend contrast-enhanced computed tomography (CECT) to evaluate vascular invasion [3], with vascular invasion determined when the lesion wraps around the blood vessels more than 180 degrees. However, the guidelines do not recommend ultrasound examination. In recent years, the development of contrast-enhanced ultrasound (CEUS) has shown significant advantages in the diagnosis of benign and malignant diseases. As a pure-blood pool contrast agent, ultrasound microbubbles can effectively simulate the movement of red blood cells inside the lesion and help visualize the relationship between the lesion and surrounding organs and blood vessels in real-time, providing a specific basis for evaluating vascular invasion. Some patients who cannot undergo CECT due to drug sensitivity, mobility difficulties, or frequent examinations can undergo CEUS.

However, the ability of CEUS to assess vascular invasion is still under investigation. EFSUMB guidelines [4] only point out that CEUS could better display the relationship between lesions and peripancreatic vessels. Previous studies have shown that color Doppler flow imaging (CDFI) has been used to diagnose vascular invasion of PDAC, but there are few comparisons with CEUS. The advantages of CEUS and CDFI and how to perform a one-stop CEUS examination of pancreatic lesions are still unclear in practice. Therefore, solving this problem can better help us apply ultrasound technology in the assessment of vascular invasion in PDAC, with the potential for further application in dynamic monitoring of chemotherapy efficacy and preoperative evaluation.

The main objective of this study was to compare the ability of CEUS and color Doppler techniques to diagnose vascular invasion of PDAC. The secondary objective was to explore how to use ultrasound technology to complete the one-stop diagnosis of pancreatic cancer and vascular invasion.

## Materials and methods

### Ethical statement

This retrospective study was reviewed and approved by our institutional review board. The requirement to obtain written informed patient consent was waived.

### Patients

**Fig. 1:**
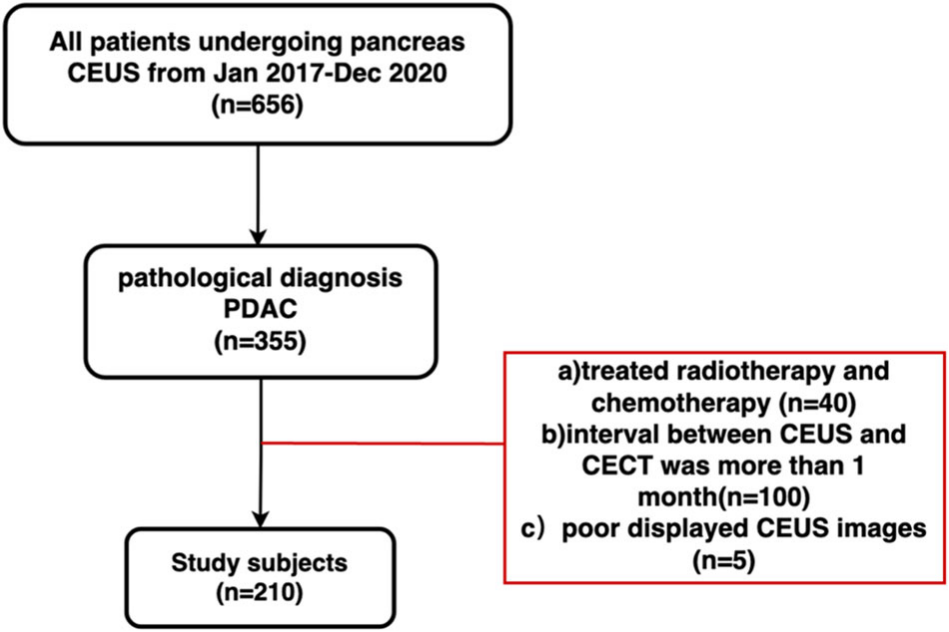
Enrollment flow chart

Between January 2017 and December 2020, we used a clinical and pathologic database to retrospectively identify 210 patients with confirmed PDAC who underwent both CEUS and CECT imaging. The inclusion criterion was as follows: (a) pathological diagnosis of PDAC based on surgical resection, percutaneous biopsy, and endoscopic ultrasound biopsy. The exclusion criteria were as follows (a) previous treatment with radiotherapy and chemotherapy; (b) interval between CEUS and CECT of more than one month; and (c) poorly displayed lesions on ultrasonography images, e.g., patients with excessive intestinal gas, poor breath-holds, etc. (Fig. 1).

### Devices and procedures

US and CEUS were performed by two radiologists (K.L. and Y.G. with 20 and 15 years of experience in abdominal diseases and CEUS, respectively). The routine CEUS protocol for the pancreas in our institution includes grayscale US, color Doppler US, and CEUS. All US examinations were performed with a Philips iU22 unit (Philips Medical Systems, Bothell, WA, USA), using a 2 to 5 MHz convex probe (2D) for the baseline examination and contrast study. A conventional two-dimensional grayscale ultrasound examination was performed on all patients in a fasting state, and the lesions’ location, size, echo, boundary, and CDFI were observed and recorded. The blood vessels of the lesions were displayed by color Doppler ultrasound, the velocity range was set at + 10–10 cm/s, and the wall filter was set at 40–50 Hz. CEUS used pulse-inversion harmonic (PIH) imaging technology, and the mechanical index (MI) was set at 0.07. Then, The US contrast agent SonoVue (Bracco, Milan, Italy) was dissolved in 5 mL of saline according to the manufacturer’s instructions. A fast bolus injection of 2.4 mL contrast agent was administered intravenously, followed by 5 mL saline. The patient was told to maintain a stationary posture to continuously observe the dynamic perfusion process of the lesion in real-time over a minimum duration of 2 min and 30 s. All enhanced dynamic images were saved in AVI format.

For CECT, a 64-row spiral CT machine was used for the initial scan, followed by an intravenous dose of iopromide (1.5 mL/kg) at a rate of 3 mL/s and a three-phase dynamic scan with scanning times of 35 s after the intravenous dose in the arterial phase, 60 s after in the portal phase and 210 s after in the delayed phase. In all cases, thin layer reconstruction was performed with a layer thickness of 1.0 mm.

### Image analysis

**Fig. 2:**
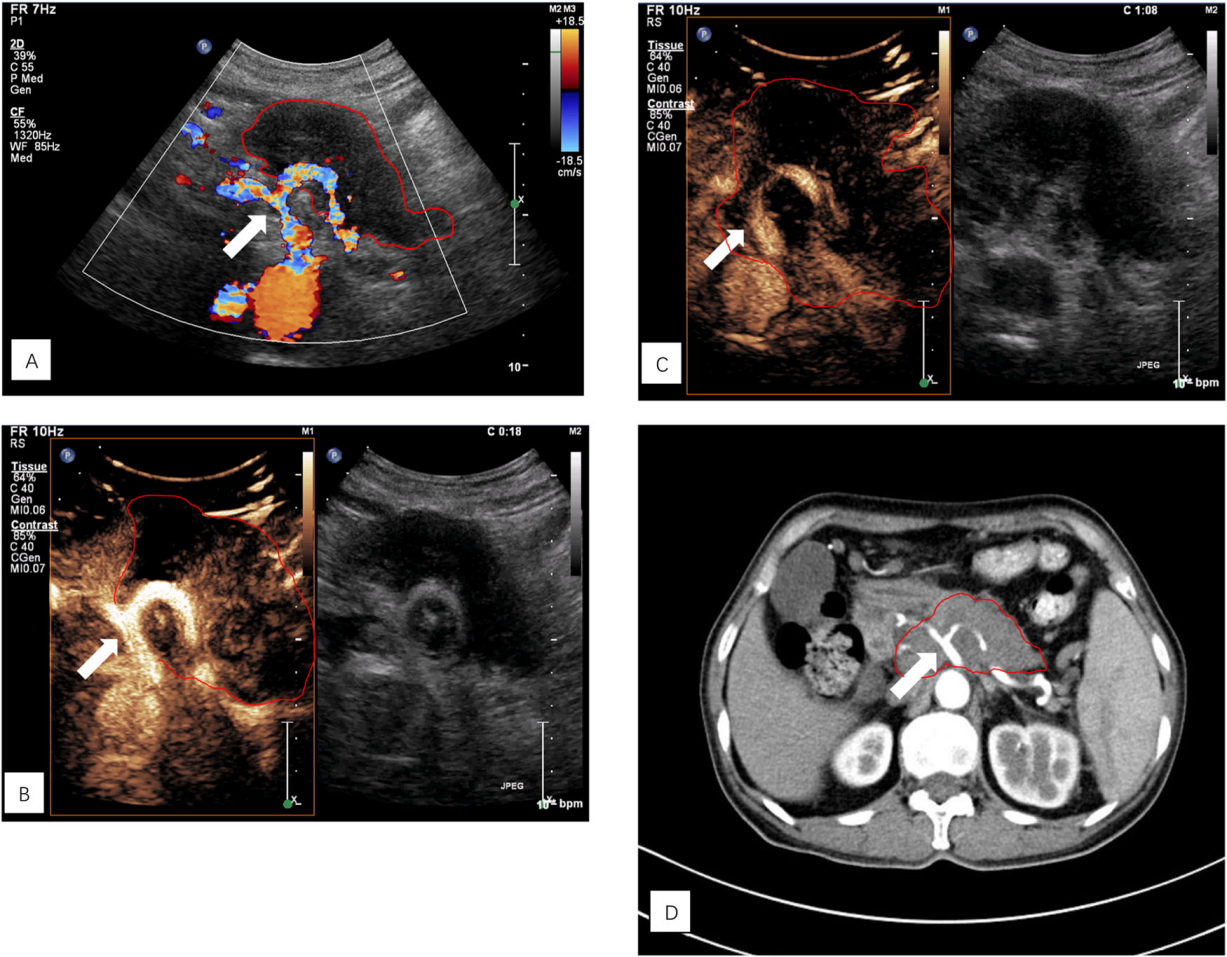
A 58-year-old female patient with PDAC located in the body of the pancreas. **A** Color Doppler US showed the relationship between the CA (arrow) and the lesion (red border), which was close to but not in contact with the CA. **B** The arterial phase CEUS scan showed the relationship between the CA (arrow) and lesion (red border), which was in contact with the CA. **C** The venous phase CEUS scans showed the relationship between the CA (arrow) and the lesion (red border), which completely surrounded the CA. **D** The arterial phase CECT scan showed that the lesion (red border) was completely wrapped around the CA (arrow)

**Fig. 3:**
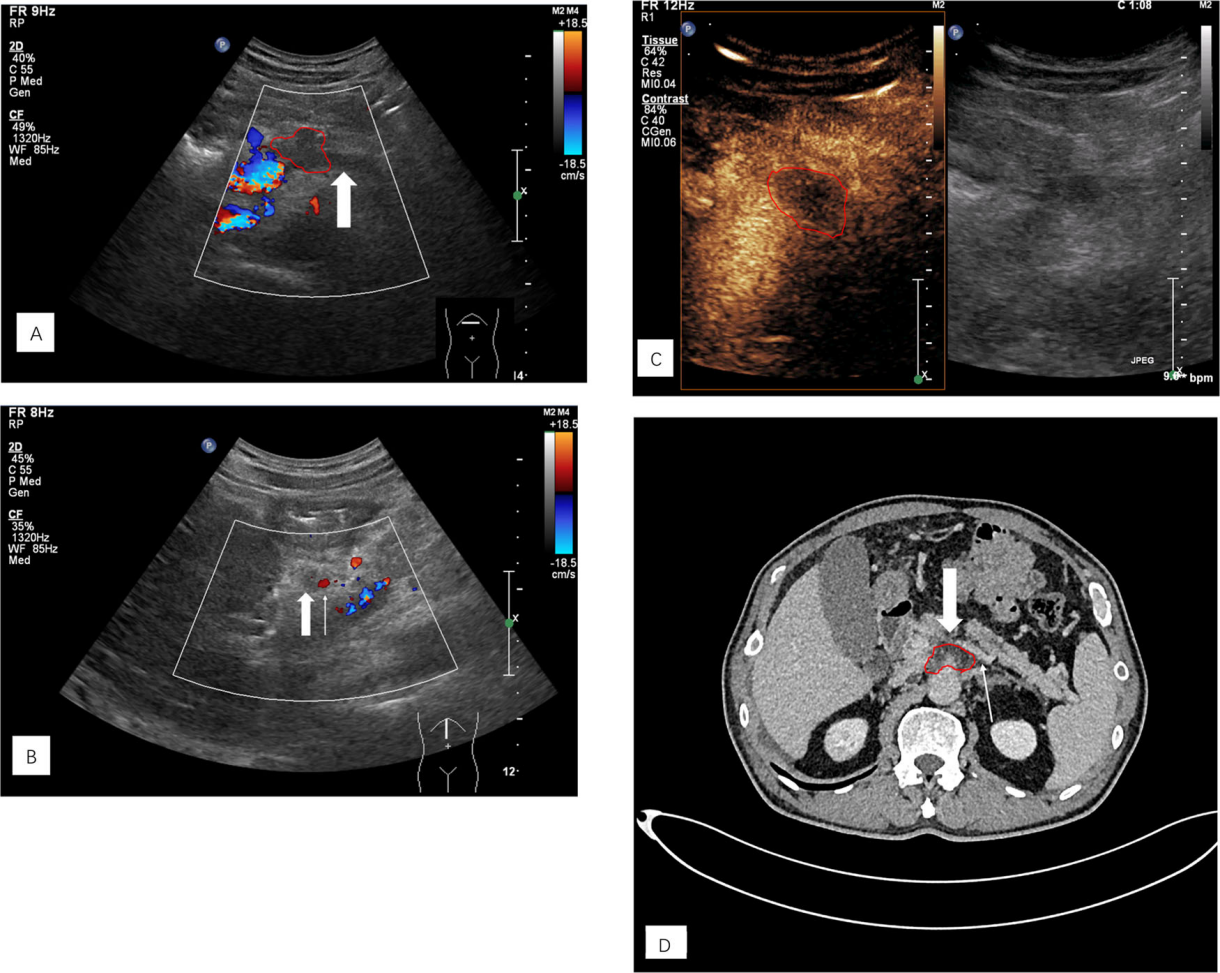
A 60-year-old male patient with PDAC located in the body of the pancreas. **A** The cross-sectional color Doppler scan showed that the lesion (red border) invaded the SPV, resulting in complete occlusion of the SPV (arrow). No blood flow signal was found on CDFI. **B** Longitudinal section of the color Doppler scan showed complete occlusion of the SPV (arrow), and no blood flow signal was observed on CDFI. The SPA could be seen beside the SPV (fine arrow). **C** On the venous phase CEUS scan, only the hypoenhanced lesion (red border) was visible, and the adjacent occluded SPV was difficult to distinguish. **D** On venous phase CECT scan, it could be seen that the SPV adjacent to the lesion (red border) was occluded (arrow), while the distal SPV was unobstructed (thin arrow)

All CEUS images were anonymized and randomly reviewed by two radiologists (W.Y.J. and X.Q.C., residents and attending physicians with 5 and 6 years of experience in abdominal diseases and CEUS, respectively). All CECT images were anonymized and randomly reviewed by two radiologists (X.Q.Z and J.H.Z., residents and attending physicians with 5 and 6 years of experience in abdominal diseases and CECT, respectively). In cases of disagreement, the two readers of each imaging method reassessed the image that yielded discrepant findings to reach an agreement. Based on the NCCN guidelines and previous studies [5–7], the tumor-vessel relationship was categorized into 2 types**:** grade 1, ≤ 180 degrees of involvement; and grade 2, > 180 degrees of involvement or any thrombosis (Figs. 2 and 3). The presence of a lesion surrounding the vessel by > 180 degrees was considered a vascular invasion. The vessels assessed include the superior mesenteric artery (SMA), superior mesenteric vein (SMV), celiac artery (CA), hepatic artery (HA). splenic artery (SPA), splenic vein (SPV), and portal vein (PV). Vascular invasion was calculated in each case, and if more than one invasion was present, the counts were accumulated separately.

In CDFI examination, the relationships between pancreatic lesions and the visible vessels listed above were determined from recorded still images. In CEUS, the relationships between pancreatic lesions and the abovementioned visible vessels were determined from recorded dynamic images. The degree of involvement between the lesion and the vessels was comprehensively judged on the arterial phase (< 30 s) and venous phase (30 s–120 s) images. The venous phase images were used to determine the actual extent of the lesion.

Finally, according to the result of the above observation of CEUS + CDFI, CECT, and NCCN guidelines, all cases were divided into resectable group, borderline resectable group, and unresectable group. In ultrasound evaluation, when there is a disagreement between CEUS and CDFI: the evaluation of arterial vessels is based on CEUS, while the venous vessels are based on CDFI.

### Interreader agreement for ultrasound

All ultrasound data (including color Doppler US and CEUS) were independently classified again according to the principles stated in the image analysis section by six radiologists with three different experience levels in reading CEUS: two residents (< 1 year, < 100 cases), two fellows (5–6 years, 3000–4000 cases), and two specialist staff members (> 15 years, > 5000 cases).

### Statistical analysis

Categorical variables are expressed as rates in percentages and absolute numbers. Continuous variables are expressed as the mean ± standard deviation. The *χ*^2^ test was used to analyze differences between categorical variables and the variance test was used to analyze differences between continuous variables. To evaluate the diagnostic performances of each modality for vascular invasion, the sensitivity (SEN), specificity (SPE), positive predictive value (PPV), negative predictive value (NPV), accuracy (ACC), and receiver operating characteristic curve (ROC) for each blood vessel were calculated, *p* < 0.05 indicated a significant difference. The Kappa test was used to evaluate interreader agreement for color Doppler US and CEUS. Agreement was considered slight (0.01–0.020), fair (0.21–0.40), moderate (0.41–0.60), substantial (0.61–0.80), or almost perfect (0.81–1.00). Statistical analysis was performed with SPSS software (version 22.0) and MedCalc software (version 20.0).

## Results

### Patient characteristics

The 210 consecutively enrolled patients included 87 females and 123 males with a median age of 60.36 years. Ninety-five (45.2%) tumors were in the head and neck of the pancreas, and 115 (54.8%) were in the body and tail. Forty-seven (22.4%) patients were believed to have potentially resectable malignancy based on the preoperative evaluation and therefore underwent surgery, 4 (1.9%) patients underwent intraoperative exploration, and 159 (75.7%) cases were deemed unresectable based on the clinical information and the overall status of the patient (i.e., vascular invasion, metastatic disease or poor surgical candidates) (Table 1).

**Table 1:** Patient characteristics

	All patients (*n* = 210)	Surgical patients	Nonsurgical patients
Age (years)	60.36 ± 9.50	60.90 ± 9.78	60.18 ± 9.44
Gender (male/female)	123 (58.6%)/87 (41.6%)	33 (64.7%)/18 (35.3%)	90 (56.6%)/69 (43.4%)
Location (head and neck/body and tail)	95 (45.2%)/115 (54.8%)	21 (56.8%)/16 (43.2%)	68 (42.8%)/91 (57.2%)
Median tumor size (cm)	4.63 ± 3.20	3.96 ± 1.28	4.84 ± 3.59

### Patients who underwent surgery and intraoperative exploration

In the 51 patients with surgical results, the lesions were located in the head and neck of the pancreas in 27 patients and the body and tail in 24 patients. The results of a comparison among color Doppler US and CEUS concerning the prediction of vascular involvement in PDAC are shown in Table 2. In PDAC of the pancreatic head and neck, there were 2 cases of arterial invasion, into the SPA and SMA. The areas under the ROC curves of color Doppler US and CEUS were all 1.00 (*p* = 1.00). There were 16 cases of venous invasion, including 5 into the SMV, 8 into the SPV, and 3 into the PV. The areas under the ROC curves of color Doppler US and CEUS were all 0.82 (*p* = 0.32). In PDAC of the pancreatic body and tail, there were 20 cases of arterial invasions, including 2 into the CA, 15 into the SPA, and 3 into the HA. The areas under the ROC curves of color Doppler US and CEUS were 0.91 and 0.96, respectively (*p* = 0.15). There were 21 cases of venous invasions, including 18 into the SPV and 3 into the PV. The areas under the ROC curves of color Doppler US and CEUS were 0.97 and 0.69, respectively. The difference was statistically significant (*p* < 0.05).

**Table 2:** Prediction of vascular invasion by color Doppler US, CEUS, and CECT in 51 patients with PDAC who underwent surgical exploration

Location	Vessel	Technique	SEN (%)	SPE (%)	ACC (%)	PPV (%)	NPV (%)	ROC	*p*
Head	Artery	Doppler	100	100	100	100	100	1.00	1.00
and		CEUS	100	100	100	100	100	1.00	
neck	Vein	Doppler	66.7	97.4	96.3	50.0	98.7	0.82	0.32
		CEUS	66.7	98.7	97.5	66.7	98.7	0.82	
Body	Artery	Doppler	85.0	97.4	94.8	89.5	96.1	0.91	0.15
and		CEUS	95.0	97.4	96.9	90.5	98.7	0.96	
tail	Vein	Doppler	95.2	98.0	97.2	95.2	98.0	0.97	< 0.05
		CEUS	38.1	100	81.9	100	79.7	0.69	

### Nonsurgical patients

**Fig. 4:**
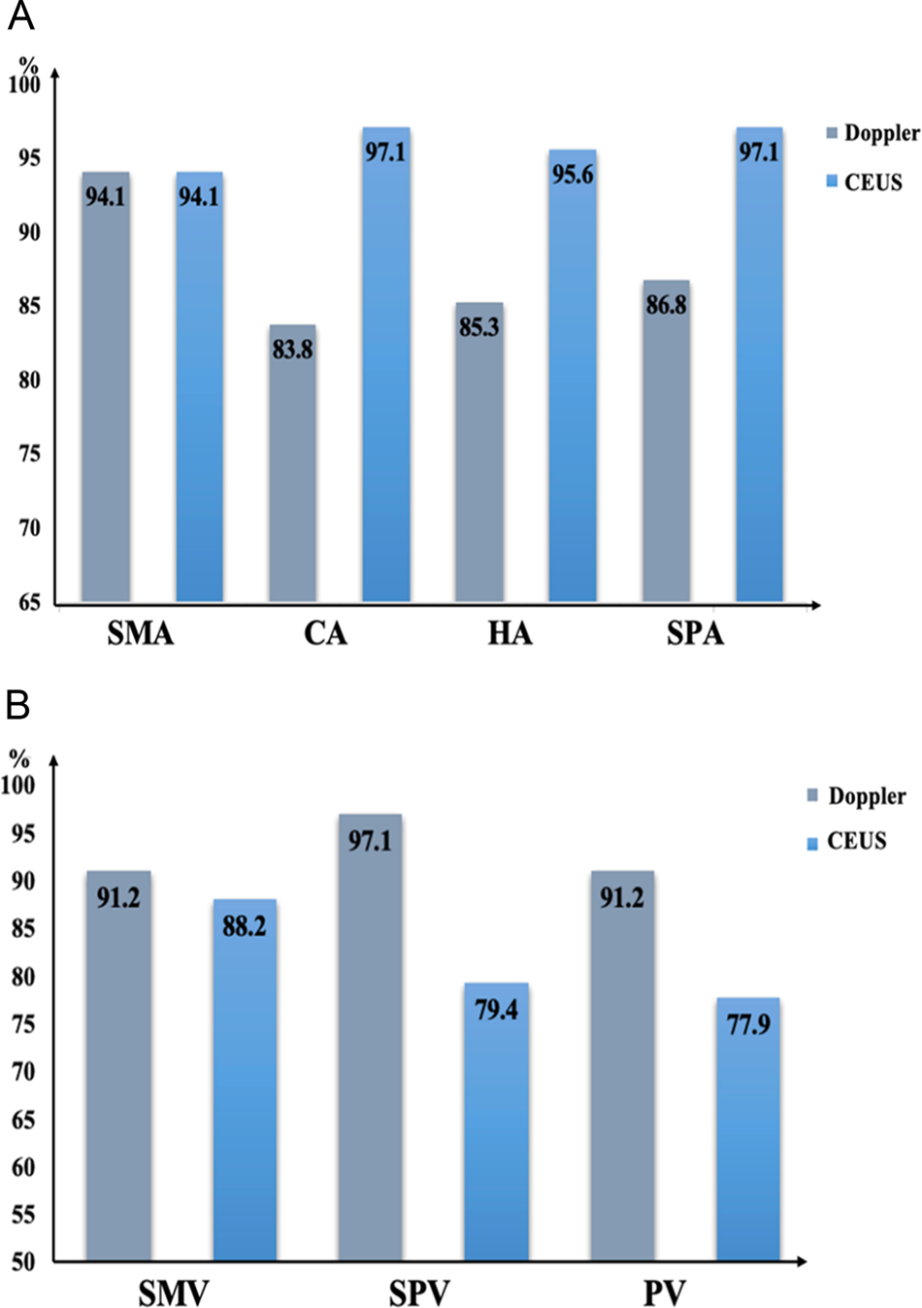
Comparison of the accuracy of color Doppler US and CEUS in diagnosing vascular invasion in a nonsurgical patient with PDAC in the pancreatic head and neck (**A**). Comparison of accuracy in identifying vascular invasion in the arterial system (SMA, CA, HA, and SPA). **B** Comparison of accuracy in identifying vascular invasion accuracy in the venous system (SMA, SPV, and PV)

**Fig. 5:**
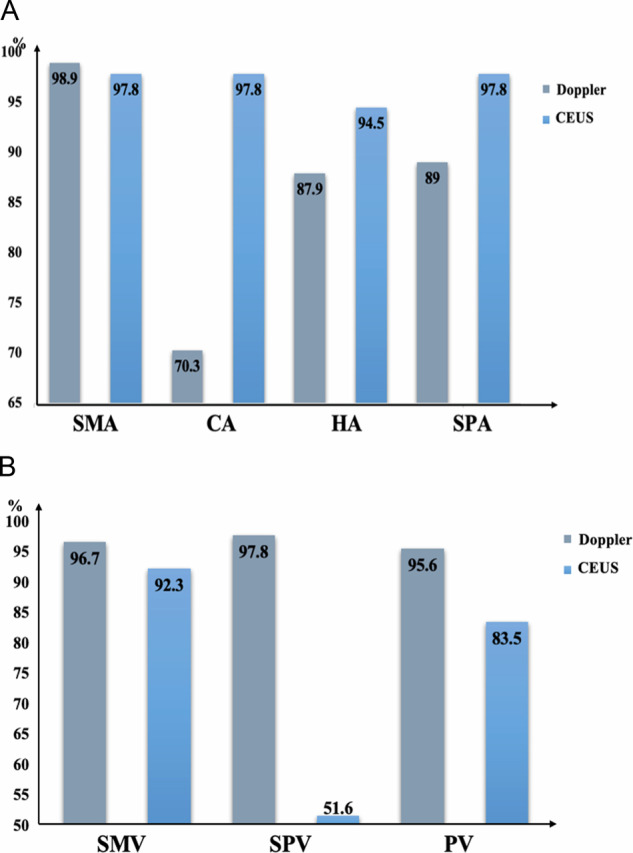
Comparison of the accuracy of color Doppler US and CEUS in diagnosing vascular invasion in a nonsurgical patient with PDAC in the pancreatic body and tail. **A** Comparison of the accuracy for identifying vascular invasion in the arterial system (SMA, CA, HA, and SPA). **B** Comparison of the accuracy for identifying vascular invasion in the venous system (SMA, SPV, and PV)

There were 159 nonsurgical patients, including 68 lesions in the head and neck of the pancreas and 91 lesions in the body and tail. Using CECT results as a reference standard, the diagnostic abilities of color Doppler US and CEUS for vascular invasion were compared, as shown in Tables 3 and 4 and Figs. 4 and 5. In PDAC of the pancreatic head and neck, there were no significant differences between color Doppler US and CEUS in the diagnosis of vascular invasion into the SMA and SMV (*p* = 0.43, *p* = 0.50). The differences for the remaining vessels were statistically significant. CEUS was superior to color Doppler US in diagnosing vascular invasion into the CA and its branches. CA-CEUS/CDFI (SEN, SPE, ACC, PPV, NPV): 89.5%/50.0%; 100%/96.0%; 97.1%/83.8%; 100%/81.8%; 96.1%/84.2%. HA-CEUS/CDFI (SEN, SPE, ACC, PPV, NPV); 83.8%/72.2%; 100%/90.0%; 95.6%/85.3%; 100%/72.2%; 94.3%/90.0%. SPA-CEUS/CDFI (SEN, SPE, ACC, PPV, NPV); 88.9%/68.4%; 100%/93.9%; 97.1%/86.8%; 100%/81.3%; 96.2%/88.5%. For invasion into venous systems, such as into the SPV and PV, the diagnostic efficiency of color Doppler US was better than that of CEUS, and the ACC of color Doppler US for diagnosing invasion into the SPV was as high as 97.1%. Similarly, in PDAC of the pancreatic body and tail, there were also differences between the two examination modalities in the diagnosis of vascular invasion into the CA, HA, SPA, SPV, and PV (*p* < 0.05). CEUS was better at assessing vascular invasion into the CA, HA, and SPA than color Doppler US, but worse than color Doppler US at assessing invasion into the SPV and PV.

**Table 3:** Comparison between color Doppler US and CEUS in diagnosing vascular invasion in 68 nonsurgical cases of head and neck PDACs

Vessel	Technique	SEN (%)	SPE (%)	ACC (%)	PPV (%)	NPV (%)	ROC	*p*
SMA	Doppler	80.0	96.6	94.1	80.0	94.1	0.88	0.43
	CEUS	80.0	98.3	94.1	88.9	96.6	0.84	
SMV	Doppler	88.4	96.0	91.2	82.8	91.2	0.92	0.50
	CEUS	86.0	92.0	88.2	94.9	79.3	0.89	
CA	Doppler	50.0	96.0	83.8	81.8	84.2	0.73	< 0.05
	CEUS	89.5	100	97.1	100	96.1	0.95	
HA	Doppler	72.2	90.0	85.3	72.2	90.0	0.81	< 0.05
	CEUS	83.8	100	95.6	100	94.3	0.92	
SPA	Doppler	68.4	93.9	86.8	81.3	88.5	0.81	< 0.05
	CEUS	88.9	100	97.1	100	96.2	0.94	
SPV	Doppler	97.4	96.6	97.1	97.4	96.6	0.97	< 0.05
	CEUS	66.7	96.6	79.4	96.3	68.3	0.82	
PV	Doppler	85.2	95.1	91.2	92.0	90.7	0.90	< 0.05
	CEUS	48.1	97.6	77.9	92.9	74.1	0.73

**Table 4:** Comparison between color Doppler US and CEUS in diagnosing vascular invasion in 91 nonsurgical patients with body and tail PDACs

Vessel	Technique	SEN (%)	SPE (%)	ACC (%)	PPV (%)	NPV (%)	ROC	*p*
SMA	Doppler	80.0	100	98.9	100	98.9	0.90	0.32
	CEUS	80.0	98.8	97.8	80.0	98.8	0.89	
SMV	Doppler	91.2	98.2	95.6	96.9	98.2	0.95	0.57
	CEUS	88.2	94.7	92.3	90.9	93.1	0.92	
CA	Doppler	46.8	95.5	70.3	91.7	62.7	0.71	< 0.05
	CEUS	93.6	100	97.8	100	93.6	0.97	
HA	Doppler	81.3	95.3	87.9	95.1	82	0.88	< 0.05
	CEUS	93.8	97.7	94.5	97.8	93.3	0.96	
SPA	Doppler	90.8	80.0	89.0	95.8	63.2	0.85	< 0.05
	CEUS	96.1	86.7	97.8	97.4	82.4	0.95	
SPV	Doppler	97.4	100	97.8	100	88.2	0.99	< 0.05
	CEUS	47.4	73.3	51.6	90.0	21.6	0.60	
PV	Doppler	84.2	98.6	95.6	94.1	95.9	0.91	< 0.05
	CEUS	26.3	98.6	83.5	83.3	83.5	0.63

### Interreader agreement between color Doppler US and CEUS for assessing vascular invasion in PDAC

For all 1470 vessels in 210 patients, the interreader agreement between radiologists of different experience levels for color Doppler US and CEUS in the assessment of vascular invasion in PDAC is shown in Table 5. Fellows and specialists had almost perfect interreader agreement (*κ*, 0.89–0.93), both between readers of the same experience levels and between readers with two different experience levels in reading color Doppler US and CEUS images. CDFI/CEUS: Re&Re 0.70/0.83, Fe&Fe 0.85/0.89, Spe&Spe 0.87/0.92, Re&Fe 0.78/0.89, Re&Spe 0.65/0.88, Fe&Spe 0.88/0.93 (Re, resident; Fe, fellow; Spe, specialist). It is worth noting that with CEUS, we found improvement in interreader agreement among residents, with *κ* improving from substantial (*κ*, 0.65–0.78) to almost perfect (*κ*, 0.83–0.89), with a statistically significant difference.

**Table 5:** Interreader agreement between color Doppler US and CEUS in assessing vascular invasion of PDAC

	Color Doppler (*κ*)	95% CI	CEUS (*κ*)	95% CI	*p*
Re&Re	0.70	0.66–0.74	0.83	0.80–0.86	< 0.05
Fe&Fe	0.85	0.82–0.88	0.89	0.87–0.91	0.29
Spe&Spe	0.87	0.84–0.90	0.92	0.90–0.94	0.47
Re&Fe	0.78	0.75–0.81	0.89	0.87–0.91	< 0.05
Re&Spe	0.65	0.61–0.69	0.88	0.85–0.91	< 0.05
Fe&Spe	0.88	0.85–0.91	0.93	0.91–0.95	0.23

Data are kappa scores. *Re* resident, *Fe* fellow, *Spe* specialist

### Evaluation of PDAC resectability by ultrasound and CECT

According to the above characteristics of CEUS and CDFI in the examination of different types of blood vessels, we integrated their respective advantages, and the arterial vessel evaluation was mainly CEUS, while the venous vessel evaluation was mainly CDFI. Resectable determination was made by evaluating the results of each blood vessel assessment for each patient, as shown in Fig. S1. Ultrasound can achieve similar performance to CECT.

## Discussion

Radical surgical resection (R0 resection) is currently the only way to improve the 5-year survival rate of patients with PDAC [8]. In the absence of distant metastasis and lymph node metastasis, the resectability of pancreatic cancer mainly depends on the presence of invasion into peripancreatic vessels. It is widely believed that ultrasonography has limitations in diagnosing pancreatic disease. However, with the development of CEUS technology, it is easier to display lesions more clearly, compensating to some extent for this deficiency. The previous results of our team and in the relevant literature [4, 9] confirm the importance of CEUS in diagnosing pancreatic lesions. However, the use of CEUS for assessing vascular invasion is still under investigation.

Invasion into the CA is a determinant of surgical treatment in patients with PDAC. Therefore, assessments of such invasion are critical. Since vascular invasion is a contraindication to surgery, the number of positive vessels in this analysis was small. As a result, color Doppler US and CEUS did not show significant differences in the evaluation of vascular invasion. This was effectively remedied in the analysis of nonsurgical patients, for whom we evaluated a total of 1113 vessels (SMA, SMV, CA, HA, SPA, SPV, and PV) in 159 patients using CECT results as a reference standard to identify diagnostic differences between color Doppler US and CEUS in the assessment of vascular invasion. Our results show that CEUS was significantly superior to color Doppler US in evaluating the CA and its branches. When invasion into the CA was present in the PDAC patients, CEUS had an ACC of up to 97.8%. We believe that the reasons are as follows: (1) the CA location is deep, and color Doppler US has a limited depth of detection, making it difficult to properly visualize the presence of invasion; and (2) there may be some occult foci around PDAC lesions, which are not shown on ultrasound grayscale images. When a contrast agent was injected, the range of the actual lesions could be displayed, and the relationship between the lesions and the CA could be accurately evaluated. Using the CA as an example (Fig. 2), the CA and its branches were seen to be rapidly bright and highly enhanced approximately 10 s after the injection of the contrast agent, in sharp contrast to the surrounding unfilled pancreatic lesions and pancreatic parenchyma. In the venous phase, CEUS can reveal the actual extent of the lesions, thus facilitating a correct diagnosis. However, on color Doppler examination, we saw a certain distance between the lesion and the CA (Fig. 2A), which could lead to misjudgment that the lesion did not invade the CA.

In the venous system, we found some interesting results that differed from the EFSUMB guideline. Our study showed that CEUS is inferior to color Doppler US in assessing the venous system as a whole, especially in the SPV, where color Doppler US can be up to 97.8% accurate in diagnosing invasion into the SPV. Taking the SPV as an example, we found it difficult for CEUS to show a wholly occluded SPV (Fig. 3). This may be explained by the fact that the venous lumen is thin and the pressure is low, so when a vascular invasion occurs, complete occlusion of the venous lumen can quickly occur. In this situation, it is often difficult to distinguish the location of the occluded vessel during CEUS imaging; even if we try to carefully determine the location of the vessel and presence of invasion into the SPV based on the vascular anatomy, the diagnostic results are still affected by the time frame of the ultrasound contrast agent. In contrast, in color Doppler examinations, the operator can correctly diagnose invasion by observing the vessel for a certain period. For the SMA and SMV, color Doppler US and CEUS did not show significant diagnostic differences because of the more superficial locations of these two vessels.

Although there are few studies on the evaluation of vascular invasion by CEUS, the studies on the assessment of vascular invasion by US and color Doppler US can provide some reference value. It was reported that US combined with color Doppler US could improve the ACC of diagnosing vascular invasion [10**–**12], with an SEN of 60%–90% and SPE and PPV of up to 90%, but the NPV was only approximately 75% [13**–**17]. In 22% of patients with pancreatic neoplasms, the color Doppler US results could modify the therapeutic strategy [18]. An article also reported US is 93% accurate in detecting PV invasion when using 3D vascular reconstruction technology [19]. Grossjohann H.S. et al [20] used CEUS to evaluate vascular invasion in PDAC, but only 49 cases of pancreatic head cancer were evaluated, and statistical analyses and evaluations of each blood vessel were not performed. In our study, CEUS, as a new technology, could display microcirculation perfusion of the target tissues and surrounding vessels and show lumen changes more clearly, making up for the limited diagnostic efficiency of color Doppler US for invasion into the CA and its branches, significantly improving the diagnostic efficiency of ultrasound technology for vascular invasion and supplying more practical information for clinical treatment.

It has been widely believed that the use of ultrasound in the examination of pancreatic lesions is limited due to several factors. However, through extensive case studies, our team concluded that the flexible use of the probe and the patient’s body position could yield higher examination satisfaction. In addition, CEUS showed the boundaries of the pancreatic lesion more clearly, making it easier to visualize and identify the lesion and effectively reducing operator dependency in ultrasonography. CEUS improves diagnostic ACC by acquiring and archiving dynamic video, which a more experienced radiologist can review after completion. Moreover, the interobserver agreement results in our study showed that CEUS significantly improved the agreement of residents with fellows and specialists compared to color Doppler US, probably because the contrast between the vessels and lesions was more pronounced on CEUS images, which helps the operator make the correct conclusion.

**Fig. 6:**
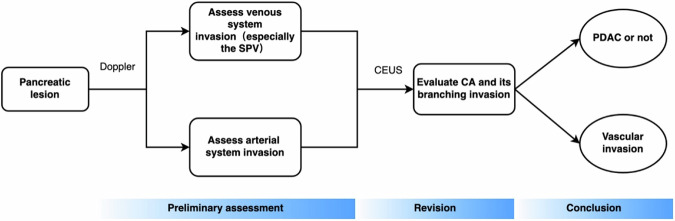
CEUS examination procedure for PDAC

CEUS is superior to color Doppler US for the assessment of vascular invasion, especially in the CA and its branches, as CEUS clearly shows the boundaries of the lesion and better demonstrates its relationship to the surrounding vessels. However, when the invasion completely occludes the vessel in the venous system, visualization is not as straightforward with CEUS as with color Doppler US. The following procedure is recommended for the CEUS evaluation of vascular invasion in PDAC (Fig. 6). First, the location of the lesion should be determined, and better ACC can be obtained by using color Doppler US to generally assess the large vessels surrounding the pancreas and specifically examine the venous system. CEUS should then be performed to determine the lesion’s nature and borders and its relationships to the large peripancreatic vessels and their branches on arterial and venous phase images. Lastly, a final diagnosis can be made based on the CEUS and color Doppler findings. Our team found that by standardizing the CEUS imaging process, the assessment of the nature of the lesion and the presence of vascular invasion could be completed in a single CEUS session, without the need for additional CEUS sessions to assess vascular invasion or increasing the medical burden on patients and society. In recent years, a large number of studies have shown [21–23] that CECT is not ideal for assessing the resectability of lesions in patients treated with chemotherapy and neoadjuvant chemotherapy. This is due to the inflammatory response and fibrosis of the tissue surrounding PDAC tumors present after neoadjuvant therapy, making it difficult for CECT to identify the tumor tissue and posing an obstacle in the accurate assessment. Our study provides a preliminary basis for CEUS to evaluate vascular invasion of PDAC, providing more information for the choice of treatment options.

Several limitations in our study should be considered. First, this study was retrospective. The results need to be validated by a prospective study with a larger sample size. Second, in this study, CECT results were used as the diagnostic criterion for vascular invasion in the group of patients who did not undergo surgery, mainly because patients with vascular invasion could not be treated surgically. However, CECT has become the method of choice for diagnosing vascular invasion, and its ACC in non-chemoradiotherapy cases is reliable. Finally, some arterial branches have anatomical variations, such as the variation rate of 0.5–5% [24] in the HA, which is also an important influencing factor in determining vascular invasion of the lesion. Although anatomical variation was not included in the study, it did not affect the diagnostic ACC of single-vessel invasion and had no significant effect on the study results. In a subsequent study, we will include this in a prospective study.

## Conclusion

In summary, CEUS has high diagnostic value in evaluating vascular invasion in patients with PDAC, especially invasion into the CA and its branches. For occlusions of the venous system, especially in the SPV, color Doppler US achieved satisfactory diagnostic efficacy. CEUS combined with color Doppler US not only provides more abundant information for the clinical diagnosis, treatment, and monitoring of PDAC but is also expected to be used as a potential complement to CECT in the evaluation of vascular invasion in PDAC patients with previous chemotherapy.

### Supplementary Information

ELECTRONIC SUPPLEMENTARY MATERIAL

## Data Availability

The data and material are available from the corresponding author, Prof. Ke Lv, upon reasonable request.

## Abbreviations

ACC: Accuracy
CA: Celiac artery
CDFI: Color Doppler flow imaging
CECT: Contrast-enhanced computed tomography
CEUS: Contrast-enhanced ultrasound
HA: Hepatic artery
NCCN: National Comprehensive Cancer Network
NPV: Negative predictive value
PDAC: Pancreatic ductal adenocarcinoma
PPV: Positive predictive value
PV: Portal vein
ROC: Receiver operating characteristic curve
SEN: Sensitivity
SMA: Superior mesenteric artery
SMV: Superior mesenteric vein
SPA: Splenic artery
SPE: Specificity
SPV: Splenic vein
