# Supplementary material for: Efficacy of color Doppler ultrasound and contrast-enhanced ultrasound in identifying vascular invasion in pancreatic ductal adenocarcinoma
Source: Insights Imaging. 2024 Jul 25;15:181. doi: 10.1186/s13244-024-01779-5 (PMC11282047; doi:10.1186/s13244-024-01779-5)

Efficacy of color Doppler ultrasound and contrast-enhanced ultrasound in identifying vascular invasion in pancreatic ductal adenocarcinoma

ELECTRONIC SUPPLEMENTARY MATERIAL

Figure S1 Evaluation of resectable pancreatic cancer by ultrasound and CECT

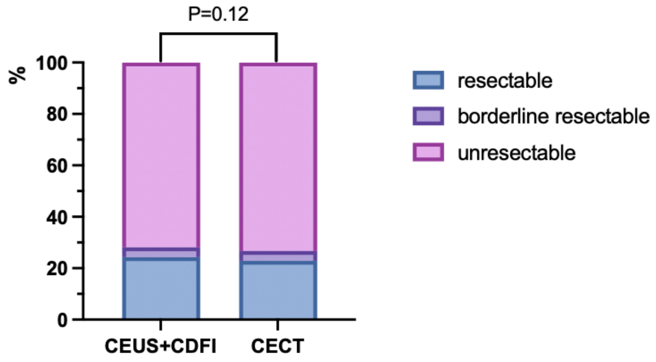

Supplement: Supplementary file 1 — ELECTRONIC SUPPLEMENTARY MATERIAL [file 13244_2024_1779_MOESM1_ESM.pdf]
